# Corrigendum to: An Innovative Approach for Facial Rejuvenation and Contouring Injections in Asian Patients

**DOI:** 10.1093/asjof/ojab024

**Published:** 2021-07-10

**Authors:** Haiyan Cui, Haiguang Zhao, Haisong Xu, Guobao Wang, Linlin Tan

In the article "An Innovative Approach for Facial Rejuvenation and Contouring Injections in Asian Patients" by Haiyan Cui, Haiguang Zhao, Haisong Xu, Guobao Wang, and Linlin Tan, that published in Volume 3, Issue 2 in *Aesthet Surg J Open Forum*, ojaa053, https://doi.org/10.1093/asjof/ojaa053 the authors included an incorrect version of Figure 2. The correct figure appears below. The authors regret this error.


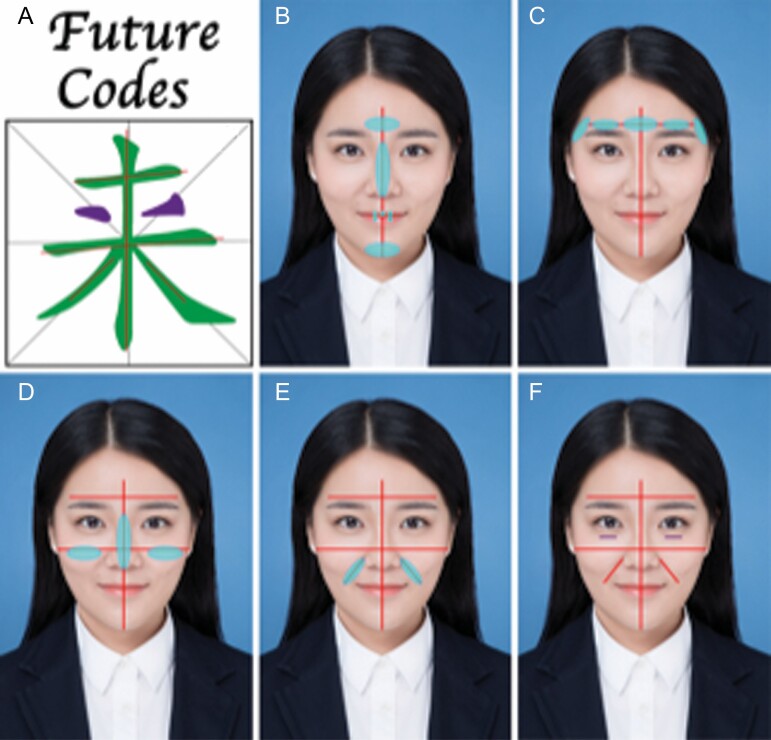


Figure 2. Dr. Cui’s “Cui Codes” design based on Chinese calligraphy. (A) 2 Chinese characters “未来,” which means “Future” in English. The concept encompasses the systematic overall design for the art of facial injection in Asians. (B) The middle line of the face (a 26-year-old female) passes through the forehead, the glabella complex, the nose, the lips, and the chin. (C) The first horizontal line passes the arch of the temper region, the eyebrow, and the glabella complex. (D) The second horizontal line passes through the cheeks or the “apple muscle.” (E) The other 2 oblique lines run along the nasolabial folds. (F) Finally, 2 nasojugular folds, tear trough, are added.

